# Mediated effects of LIFE4YOUth—a mobile health intervention for multiple lifestyle behavior change among high school students in Sweden: findings from a randomized controlled trial

**DOI:** 10.1186/s12889-025-22097-5

**Published:** 2025-03-07

**Authors:** Linnea Hedin, Anna Seiterö, Joel Crawford, Marcus Bendtsen, Marie Löf

**Affiliations:** 1 Department of Medicine Huddinge, Karolinska Institutet, Stockholm, 141 83 Sweden; 2 Allied Health Professionals, Karolinska University Hospital, Stockholm, Sweden; 3 Department of Health, Medicine, and Caring Sciences, Linköping University, Linköping, Sweden

**Keywords:** Prevention, Mediator, mHealth, eHealth, Lifestyle behaviors

## Abstract

**Background:**

Digital interventions have been shown to improve adolescents’ health behaviors. However, little is known about the mechanisms of change related to multiple risk behaviors in this population. This study aimed to estimate the natural direct and indirect effects of a digital intervention for multiple health behavior change in high school students.

**Methods:**

This was a secondary analysis of mediated effects of a digital intervention based on data from a randomized controlled trial among high school students in Sweden. Participants were classified as being at risk with respect to having at least one health behavior among lack of physical activity, unhealthy diet, alcohol consumption, or smoking. The digital intervention comprised of weekly monitoring and feedback of health behaviors together with content on strategies for behavior change. The comparator was referral to a national website where health information was available. Primary outcomes were self-reported physical activity, diet, alcohol consumption, and smoking at 4 months post-randomization. Based on a counterfactual framework, three potential mediating factors were assessed: importance, knowledge of how to change (know-how), and confidence.

**Results:**

Between September 2020 and June 2023, 756 high school students were recruited. The estimated indirect effect on moderate-to-vigorous physical activity via the mediating factors was 5.2 min (95% CoI = -8.6; 19.9) while the estimated direct effect was 76.3 min (95% CoI = 19.4; 134.2). For fruit and vegetable consumption, the estimated indirect effect was 0.04 daily portions (95% CoI = -0.01; 0.1), and the estimated direct effect was 0.19 daily portions (95% CoI = -0.08; 0.45). No marked mediated effects were observed concerning alcohol- or sugary-drinks-intake, and smoking.

**Conclusions:**

The observed intervention effects of increased physical activity and fruit and vegetable intake could only to a small extent be explained by increased confidence and know-how. To further understand the mechanisms of health behavior change, future studies should explore other potential mediators and evaluate different strategies for how to best assess and incorporate psychosocial mediators in multiple lifestyle behavior interventions for adolescents.

**Trial registration:**

Prospective registration in the ISRCTN database 20 May 2020 (ISRCTN34468623).

**Supplementary Information:**

The online version contains supplementary material available at 10.1186/s12889-025-22097-5.

## Introduction

Health risk behaviors, in terms of physical inactivity, unhealthy dietary intakes, smoking, and excessive alcohol consumption, are closely related to non-communicable diseases such as cardiovascular diseases, cancer, respiratory disease, and type II diabetes [1–3]. National surveys in Sweden demonstrate that there is a need of promoting increased physical activity (PA) and healthy dietary habits among children, and that adolescents have less healthy dietary intakes and lower PA compared with younger children [4–6]. Although smoking, and to a lesser degree alcohol consumption, have decreased among young people during recent years, harmful use of alcohol and smoking are still highly prevalent among adolescents [7, 8]. Since adolescence is a critical phase to establish long-term healthy behaviors that track into adulthood, actions that target improved health behaviors are specifically important in this age group.

Digital behavior change interventions, i.e., programs delivered through websites or mobile phones, offer accessible support for improved health behaviors, and evidence suggests that digital interventions may be effective means for improving adolescent health outcomes, including physical activity [9, 10], diet [11, 12], alcohol consumption [13], and smoking [14, 15]. Nevertheless, risk behaviors in adolescents are likely to occur in clusters rather than as isolated events [16]. Findings from a systematic review and meta-analysis of 16 school-based digital interventions, targeting multiple risk behaviors, show small short-term improvements of physical activity and fruit- and vegetable- intake. While these results show promise for intervention efforts, the authors highlight a need for more high-quality trials and additional research to better comprehend the mechanisms of change related to multiple risk behaviors in adolescents [17].

To address this issue, we conducted a randomized controlled trial (RCT) for estimating the effectiveness of a digital intervention, named LIFE4YOUth, targeting multiple health behaviors (PA, diet, alcohol, and smoking) among high school students in Sweden. The findings indicate that the intervention had a small effect on increased daily fruit- and vegetable-consumption and weekly physical activity on a moderate-to-vigorous intensity level. For the other behaviors, the evidence for an effect was weaker. These findings were based on groups of students with specific risk profiles at baseline, e.g., the effects of the intervention on physical activity were estimated among those who had physical activity below national guidelines at baseline [18].

**Fig. 1:**
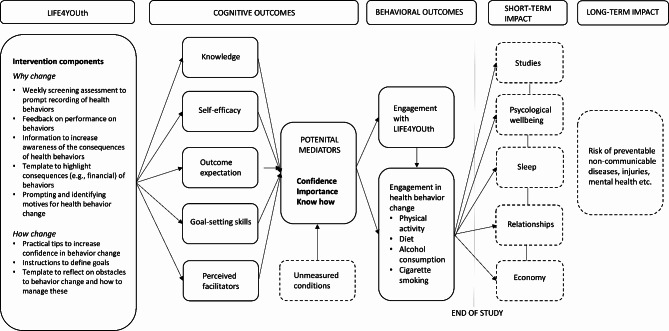
Description of the digital intervention content, behavioral factors, and mediators

The evaluated digital intervention, LIFE4YOUth, was based on current best practice on behavior change and health promotion, and the theoretical foundation stem from social-cognitive models of health and behavior change theories [19, 20]. The intervention was designed to increase behavioral intention, address environmental constraints, and improve skills necessary to perform the behavior—factors that are central in social cognitive theory models and that have been applied and evaluated in health research the last 70 years [21]. We hypothesized that promoting these factors would influence three mediators of change: (1) participants’ confidence in changing behavior to create healthy lifestyle habits [22], (2) high school students’ knowledge about how to implement actions for adopting healthy behaviors (know-how) [21, 23] and (3) how important it was for the participants to change behavior [22, 24, 25]. A description of the intervention components and their hypothesized relation with behavioral factors and potential mediators is presented in Fig. 1.

To enable more effective behavior change interventions, it is important to dismantle observed effects in trials to understand the underlying mechanisms behind the changed behavior [26–28]. To the best of our knowledge, only one previous study has evaluated potential mediators (in terms of knowledge, behavioral intentions, self-efficacy, and self-control) of a digital health intervention for multiple behavior change among adolescents [29]. To further explore potential mediators for multiple health behavior change among adolescents, this study specifically aimed to estimate the degree to which confidence, know-how, and importance mediated the effects of the LIFE4YOUth intervention on individual health behaviors.

## Methods

This study was nested in a 2-arm parallel group (1:1) single blind RCT of LIFE4YOUth with the primary objective of assessing the total effect of the intervention on PA, consumption of fruit and vegetables, sugary drinks, alcohol intake, and smoking. The study was part of the MoBILE research program which aimed to study digital behavior change interventions across the lifespan [30]. To facilitate the estimation of mediated effects, we included measures of potential mediating factors at baseline and multiple follow-up intervals. The study was approved by the Swedish Ethical Review Authority on 22 July 2020 (Dnr 2019–03813 and Dnr 2020–03538) and prospectively registered in the ISRCTN database 20 May 2020 (ISRCTN34468623). A research protocol has been published [31], following the SPIRIT guidelines [32]. This study follows the guidelines for mediator analysis reporting according to the AGReMA statement [33].

### Participants, recruitment, randomization, and blinding

A total of 403 high schools, across Sweden, recruited participants to the trial, between September 2020 to June 2023. Recruitment was done through printed advertising (posters and leaflets), digital advertising (e-mail, school website, app), and researchers advertising at the schools. Financial incentives in terms of gift cards for 100 SEK (approximately 9 USD) were awarded to participants who recruited 5 peers. Students interested in trial participation sent a text message to a dedicated telephone number. In response, a text message was received, which included a hyperlink to a webpage that contained trial information and informed consent materials. Individuals providing informed consent were immediately asked to complete the baseline questionnaire (Appendix A), which was also used to assess trial eligibility. To be eligible, the students needed to have at least one unhealthy lifestyle behavior in terms of the following conditions:

- **Weekly alcohol consumption**: Consumed 10 or more standard drinks of alcohol the past week. A standard drink of alcohol is in Sweden defined as 12 g of pure alcohol.
- **Heavy episodic drinking**: Consumed 4 or more standard drinks of alcohol on a single occasion at least once in the past month.
- **Fruit and vegetables**: Consumed less than 500 g of fruit and vegetables on average per day the past week.
- **Sugary drinks**: Consumed 2 or more units of sugary drinks the past week. This was an amendment from the study protocol (≥ 3 units) due to limitations with the questionnaire response options. One sugary drink unit is defined as approximately 33 cl.
- **Moderate to vigorous physical activity: Spent** less than 420 min on moderate to vigorous physical activity in the past week (i.e., approximately 60 min per day).
- **Smoking**: Having smoked at least one cigarette the past week.

Since the trial information and the intervention was in Swedish and delivered to participants’ mobile phones, students who could not comprehend Swedish or did not have access to a mobile phone were excluded.

Those eligible for participation were randomly allocated (1:1), using block sizes of 2 and 4, to either the control or intervention condition. Randomization was automatically conducted by the backend system and all sequences were computer generated. The trial was single blinded with research staff not aware of participant allocation.

### Interventions

Participants allocated to the control group were advised that they would get later access to the intervention and to visit a national website with general lifestyle and health information (https://www.1177.se/liv--halsa/).

**Fig. 2:**
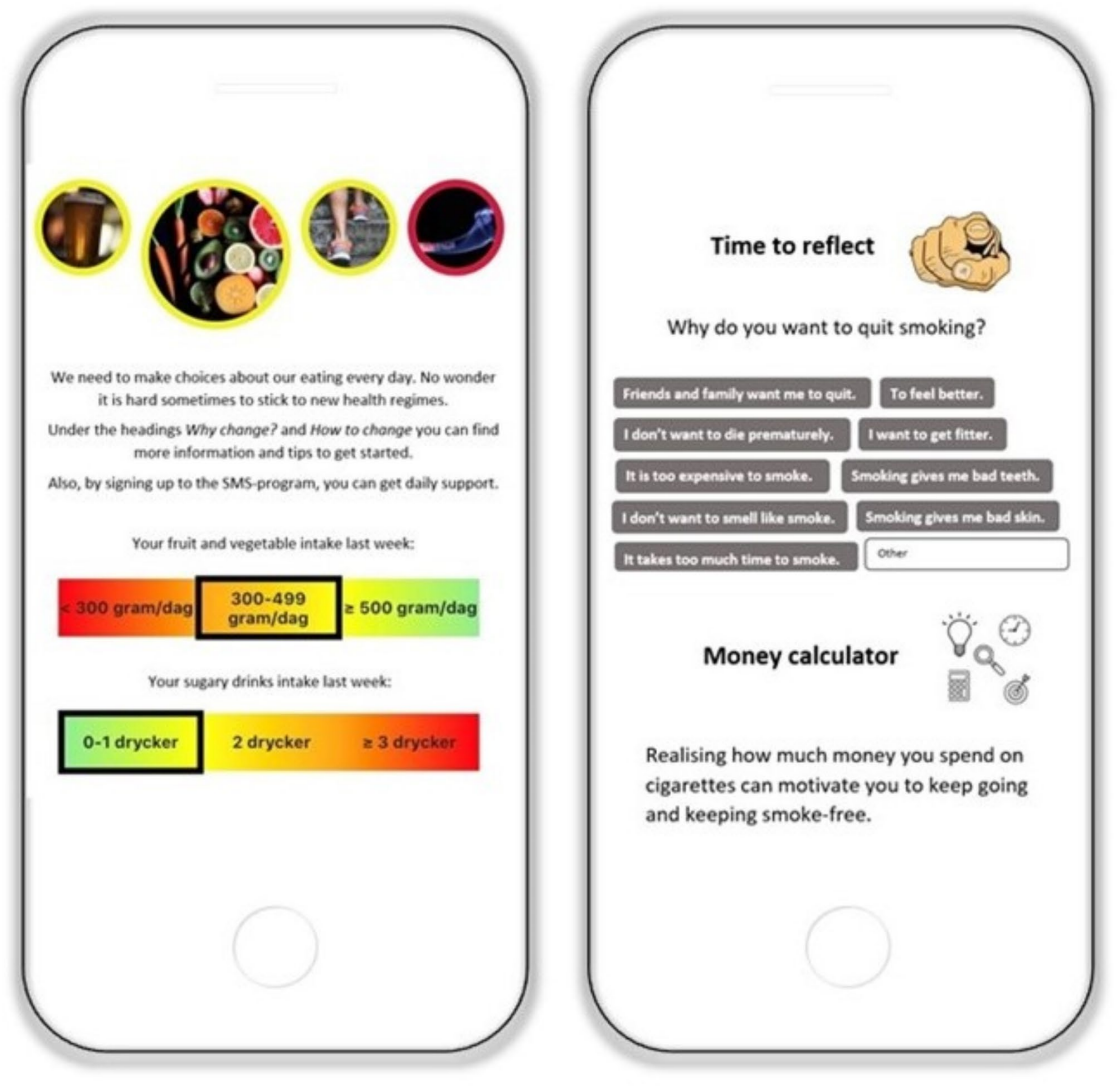
Screenshot from the LIFE4YOUTH intervention

Individuals randomized to the intervention condition were given immediate access to the digital intervention, for 16 weeks. In brief, the intervention was aimed at promoting increased physical activity, a healthy diet, reduced alcohol consumption, and smoking cessation. Participants received a text message each week including a brief screening on all four health behaviors, followed by feedback of screening results in relation to national guidelines (Fig. 2). Further, the digital intervention consisted of a personal interactive dashboard with four modules—one for each lifestyle behavior. Participants could choose to work with one module at a time or with multiple health behaviors simultaneously and each module consisted of two components: (1) content on *Why* to change a behavior and (2) *How* to change a behavior. In addition to the module components and the weekly feedback, all participants could ask for additional support in terms of automated text messages and personal reminders. See Bendtsen et al., (2021) for full details.

### Outcomes and measures

#### Mediators

Three potential mediating factors were assessed: importance, know-how, and confidence. According to several behavioral change theories [24, 25, 34] these factors are closely related, and central for changing a behavior. Importance is reflective of a person’s beliefs and attitudes towards a behavior, i.e., the motivation to change a behavior is dependent on how important the aspired behavior is to the individual [24, 34]. Know-how is related to a person’s control beliefs in terms of individual beliefs about having knowledge and skills about how to change a behavior [24]. Confidence, or self-efficacy, is reflective of a person’s capability to perform a behavior and is a cornerstone in behavioral change theories—foremost social cognitive theory [34].

The three mediator factors were assessed at baseline and at the 2- and 4-month follow-up intervals by the following questions: “How important do you think it is to improve your lifestyle or sustain your healthy behaviors?”, “How well do you know how to change your lifestyle?”, and “How confident are you that you will be able to change your lifestyle or sustain your healthy behaviors?”. The participants responded on a 10-point scale where 1 was equal to “Not at all” and 10 corresponded to “Very important/Very well/Very confident”. To reduce participant burden, as a strategy to increase retention, validated, more extensive measures of these three factors were not included in this trial. However, the items were based on single-item importance and confidence rulers which have seen a lot of empirical validation [35], focusing on a general assessment rather than a specific time interval such as the coming week or month. Further, the questions have been used in previous evaluations of mediating factors for behavior change [27, 28].

#### Behavioral outcomes

The primary outcomes of the LIFE4YOUth intervention were assessed at baseline and 2- and 4- months after randomization (Appendix A). They covered four areas: PA, diet, alcohol, and smoking. A modified version of questionnaires published by the National Board of Health and Welfare in Sweden, was used for questions on PA and diet [36].

- The outcome of *Moderate to vigorous physical activity (MVPA)*, consisted of summing the results of two questions regarding time spent on PA the past week in a moderate and vigorous intensity respectively.
- For *weekly intake of fruit and vegetables* participants reported how many portions (100 g) of fruit and vegetables (two questions, one for each topic) they consumed daily, on average, the past week.
- *Consumption of sugary drinks* was assessed by asking how many units (33 cl corresponding to 1 standard can) of sugary drinks the participants drank the past week.
- *Weekly alcohol consumption* was measured by asking participants about the number of standard drinks of alcohol they consumed in the last week and for *frequency of heavy episodic drinking* participants reported how many times they consumed more than four standard drinks of alcohol on one occasion in the past month. Both questions are included in the core outcome set for brief alcohol interventions [37, 38].
- *Smoking cessation* was assessed by participants responding to a binary question about four week point prevalence of smoking abstinence (no cigarettes the past week), a question suggested by the Society of Research on Nicotine and Tobacco [39].

#### Follow-up procedures

At 2- and 4-months post-randomization, participants received a text message with a hyperlink to questionnaires. For those not responding, two reminders were sent two days apart. If there was still no response, we called the participants to collect responses. A maximum of five attempts to reach the participants over the phone were made.

#### Effects of interest

In contrast to the primary results from the trial [31], which included participants with increased risk behavior at baseline in the analysis of each behavioral outcome, this prespecified analysis of mediators took a universal approach including all participants in all analyses. For example, when analyzing total and mediated effects on weekly alcohol consumption, all participants were included, irrespective of their baseline consumption patterns.

In this study we aimed to estimate the natural direct and natural indirect effects [40] of the intervention on each primary outcome with respect to each hypothesized mediating factor. We further aimed to estimate the total effect of the intervention on primary outcomes at the 4-month follow-up interval including all participants, regardless of baseline risk behaviors. The natural direct effect can be explained as the anticipated change in outcome prompted by switching from the control group to the intervention group while the mediating factor is kept constant at the same value it would have had if changing groups had not taken place. On the contrary, the natural indirect effect can be explained as the anticipated change in outcome prompted by being in the control group but changing the mediating factor to the value it would have had if a switch to the intervention group had been made. Based on these definitions, this study aimed to estimate:

- The total 4-month effect of the intervention on multiple health behaviors including all participants regardless of presence or absence of risk behaviors at baseline.
- The natural direct effect of group allocation on multiple health behaviors at 4 months post-randomization.
- The natural indirect effect of group allocation through mediating factors at 2- months post-randomization on multiple health behaviors assessed at 4- months post-randomization.

**Fig. 3:**
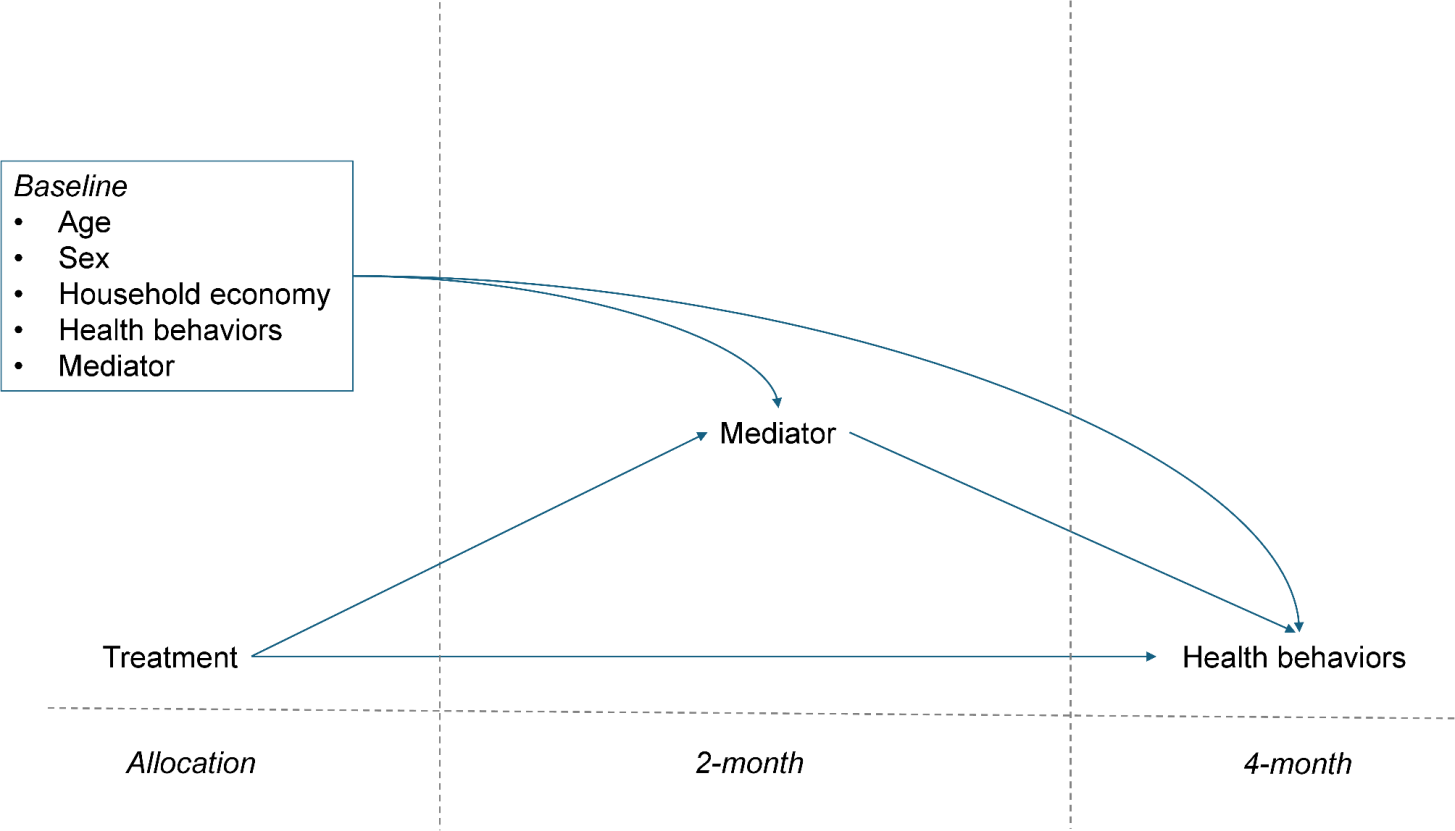
Causal model representing baseline characteristics, treatment, mediators, outcomes

Estimation of effects were pre-specified in the trial protocol as secondary objectives, following the primary total effect of the intervention [31]. Estimation of natural direct and indirect effects were based on causal models, including each mediating factor individually and in a single model including all three mediating factors simultaneously (Fig. 3). The causal models were constructed with the assumption that confounding among mediators and outcomes were removed when accounting for baseline characteristics. When interpreting the findings, it should be noted that this assumption cannot be tested.

#### Statistical methods

Participants were analyzed in their allocated group (intention-to-treat). Missing data were handled with both available data analysis and with sensitivity analyses where missing data were imputed using multiple imputation with chained equations. A counterfactual framework, following Pearl’s mediation formulas [40], was used for the analyses. Bayesian inference, with standard normal priors, was used for estimating the parameters of the models [41]. The medians of posterior distributions were used as point estimates, presented with 95% compatibility intervals (CI) defined by the 2.5% and 97.5% percentiles of the posterior distribution.

Alcohol consumption and intake of sugary drinks were modelled using negative binomial regression and linear regression was used for daily intake of fruit and vegetables, as well as MVPA in minutes per week. Logistic regression was used for point prevalence of smoking abstinence. Treatment-mediator and treatment-mediator-outcome were modelled using linear regression with mediator measures standardized. All models were adjusted for sex, age, family’s economic situation, and the baseline value of the mediator variable.

#### Attrition analyses

Attrition analyses investigated if responders and non-responders differed systematically with respect to baseline characteristics and among study groups. We used logistic regression with and without an interaction for group allocation and estimated the models with Bayesian inference [41]. To account for the large number of covariates, Cauchy priors were used for coefficients with a normal hyperprior for the scale parameter [42].

As a secondary attrition analysis, we estimated the total effect of the intervention at the 4-month follow-up interval among participants with available 2-month mediation data. Meaningful differences between estimates using all available 4-month data and only for those who also had 2-month mediator data may indicate systematic differences between those with and without mediation data.

#### Sample size

No power calculation was conducted for the mediated effects estimated in this study. A detailed description of the power calculation for the parent trial can be found in the study protocol [31].

## Results

**Fig. 4:**
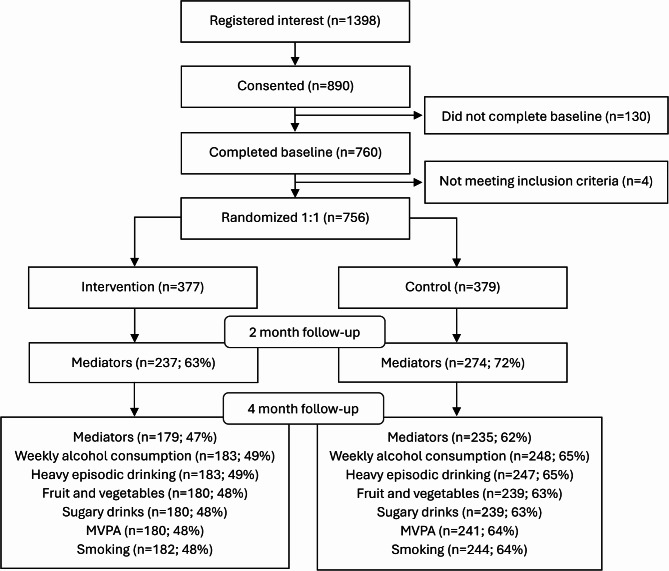
CONSORT participant flowchart

From September 14th, 2020, to June 14th 2023, a total of 1398 students showed interest in participating in the trial, of which 890 (64%) provided informed consent. Eligible individuals that completed baseline questionnaires (*n* = 756, 54%) were randomized to either the intervention (*n* = 377) or the control group (*n* = 379). The participants were 15–20 years old and had a mean (SD) age of 17.1 (1.2) years. Baseline characteristics are presented in Table 1, and a CONSORT flowchart including response rate for analyzed outcome measures is presented in Fig. 4.

**Table 1:** Baseline characteristics of randomized participants

	Total (*n* = 756)	Intervention (*n* = 377)	Control (*n* = 379)
Age, median (quartiles)	17 (16;18)	17 (16;18)	17 (16;18)
Girls, *n* (%)	520 (69)	265 (70)	255 (67)
Economy,^a^*n* (%)			
Very good	246 (33)	112 (30)	134 (35)
Average	447 (59)	232 (62)	215 (57)
Not very good	56 (7)	30 (8)	26 (7)
Poor	7 (1)	3 (1)	4 (1)
Parents education, *n* (%)			
Primary	52 (7)	25 (7)	27 (7)
Secondary	261 (35)	131 (35)	130 (34)
Tertiary	443 (59)	221 (59)	222 (59)
Region of birth, *n* (%)			
Nordic	652 (86)	326 (86)	326 (86)
Non-Nordic	104 (14)	51 (14)	53 (14)
Parents region of birth *n* (%)			
Nordic	564 (75)	275 (73)	289 (76)
Non-Nordic	192 (25)	102 (27)	90 (24)
Lifestyle outcomes, median (quartiles)			
Daily fruit and vegetable intake^b^	1 (0.7;2)	1 (0.7;2)	1 (0.7;2)
Sugary drinks^b^	2 (1;5)	2 (1;5)	2 (1;5)
Minutes in MVPA^b^	240 (75; 465)	240 (90;465)	240 (60;458)
Alcohol consumption^b^	0 (0;2)	0 (0;1)	0 (0;2)
Heavy episodic drinking^c^	0 (0;2)	0 (0;2)	0 (0;2)
Smoking^d^, *n* (%)	140 (19)	68 (18)	72 (19)
Mediators, median (quartiles)			
Importance^e^	7 (5;9)	7 (5;9)	7 (5;9)
Confidence^f^	6 (5;8)	6 (5;8)	6 (5;8)
Knowledge^g^	6 (5;8)	6 (5;8)	6 (4;8)

^a^ Self-reported estimate

^b^ Consumed frequency the past week

^c^ Frequency the past month

^d^ Smoking the past four weeks

^e^ Defined as “How important do you think it is to change your lifestyle?” on a 10-point Likert scale

^f^ Defined as “How confident are you that you will be able to change your lifestyle?” on a 10-point Likert scale

^g^ Defined as “How well do you know how to change your lifestyle?” on a 10-point Likert scale

MVPA; moderate-to-vigorous physical activity

### Outcomes and estimates

Mediators were collected from 68% of the participants at two months follow-up with responses from 72% (*n* = 274) in the control group and 63% (*n* = 237) in the intervention group. At 4 months, the mediator response rate decreased to 62% (*n* = 235) and 47% (*n* = 179) in the control- and intervention- group respectively. Approximately 65% in the control- and 50% in the intervention- group responded to the questions about behaviors at 4 months (Fig. 4). Attrition analyses showed no clear associations between baseline characteristics and non-response to either 2-month mediators or 4-month behavioral outcomes regardless of group allocation. The total intervention effect on smoking cessation was somewhat higher in participants with available 2-month mediation data. For all other behavioral outcomes at 4 months the intervention effect was similar for those with and without mediation data at 2 months (Appendix B).

### Effects of intervention and mediator outcomes

**Fig. 5:**
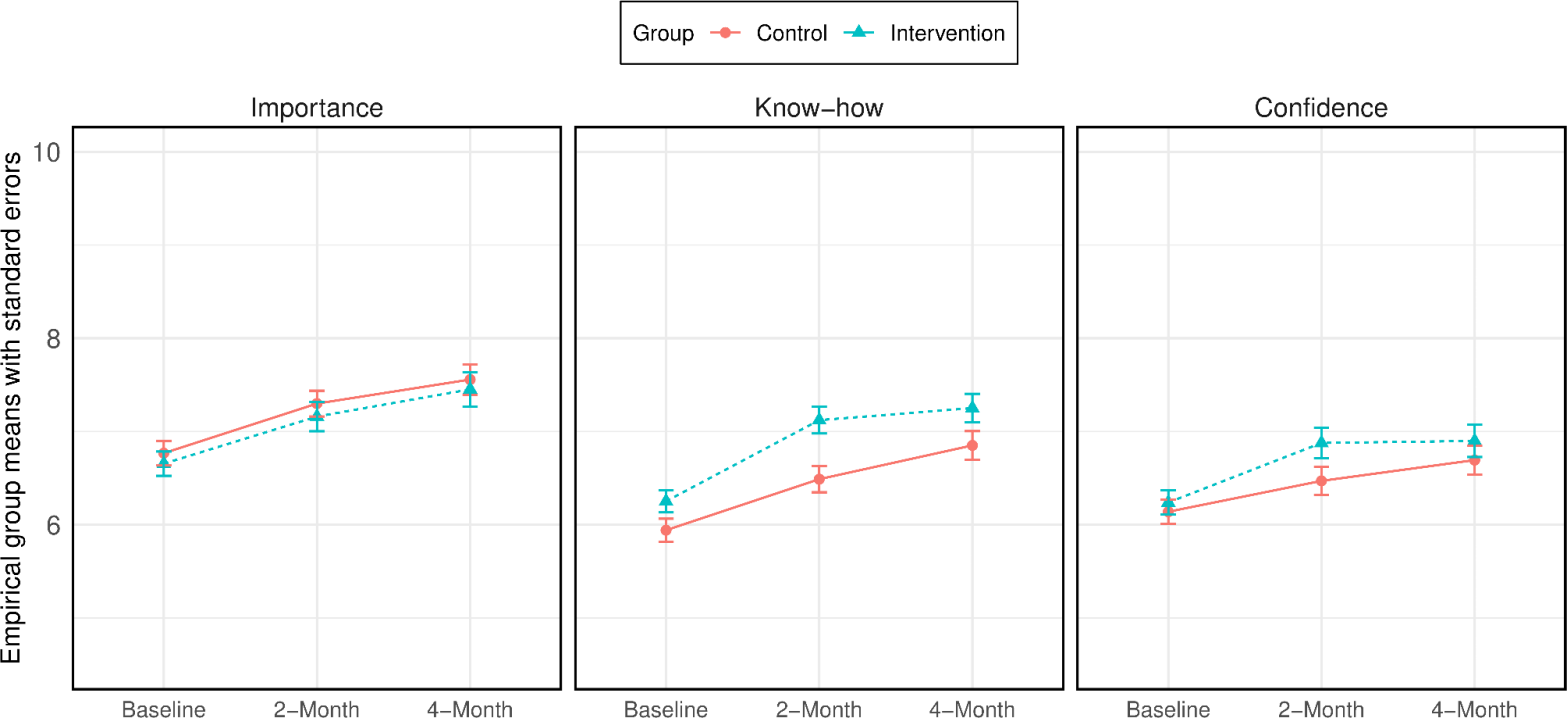
Empirical means and standard errors of mediator measures at baseline, 2- and 4-months

Mean and standard errors for the mediator measures assessed at baseline and at each follow-up interval are presented in Fig. 5. Table 2 presents an estimate of standardized effects of the intervention on mediators for available and imputed data. The baseline mediator measures were similar in the intervention- and control group at baseline with approximate mean values of 6 points for confidence and know-how and 6.5 points for importance. All mediators increased slightly over time by between 0.5 and 1.0 points at 4-months follow-up (Fig. 5). There was high probability of an intervention effect on confidence in the intervention group at 2 months follow-up. The effect remained at 4 months although it was a slightly lower effect with a somewhat lower probability of effect. Further, the results show a high probability of an effect at 2- and 4- months on know-how in the intervention group. The results further indicate that there was no observed effect of the intervention on importance. The findings were similar for the available and the imputed data (Table 2).

**Table 2:** Estimate of standardized effects of the intervention on mediator factors at 2- and 4-months

Available data	2-Month^1^		4-Month^2^	
	Est. (95% CI)	Pr.	Est. (95% CI)	Pr.
**Confidence**				
Intervention vs. Control	0.165 (0.002; 0.33)	97.6%	0.102 (-0.082; 0.291)	86.1%
**Importance**				
Intervention vs. Control	-0.055 (-0.218; 0.11)	74.4%	-0.03 (-0.218; 0.156)	62.3%
**Know-how**				
Intervention vs. Control	0.228 (0.07; 0.384)	99.8%	0.156 (-0.017; 0.329)	96.2%
**Imputed data**	**2-Month** ^**3**^		**4-Month** ^**3**^	
	**Est. (95% CI)**	**Pr.**	**Est. (95% CI)**	**Pr.**
**Confidence**				
Intervention vs. Control	0.158 (-0.006; 0.321)	97.0%	0.117 (-0.069; 0.294)	88.8%
**Importance**				
Intervention vs. Control	-0.076 (-0.235; 0.083)	82.7%	-0.018 (-0.19; 0.157)	58.1%
**Know-how**				
Intervention vs. Control	0.217 (0.056; 0.372)	99.6%	0.167 (0.001; 0.336)	97.6%

^1^Available data at 2 months; intervention *n* = 237, control *n* = 274. ^2^Available data at 4 months; intervention *n* = 179, control *n* = 235. ^3^Imputed data at 2- and 4- months; intervention *n* = 377, control *n* = 379

Est.– Median of the marginal posterior distribution of adjusted standardized effects

CI– Compatibility interval (defined by the 2.5% and 97.5% percentiles of the posterior distribution

Pr.– Proportion of the posterior distribution above or below the null, in the direction of the point estimate

### Total intervention effects

The total effect of the intervention on behaviors at 4 months are presented in Table 3. Based on available data, daily fruit and vegetable consumption was 19 g (95% CI -0.05 to 0.42) higher in the intervention group, with a probability of 94% that the intervention had any effect on fruit and vegetable intake. Corresponding results based on imputed data were 16 g (95% CI -0.06 to 0.39) higher in the intervention group with a probability of 92.5% for any intervention effect. Further, there was no marked evidence of any effect on weekly intake of sugary drinks. For physical activity, the results show strong evidence of an intervention effect, with a posterior probability of > 98% for both available and imputed data. The intervention group had a higher MVPA of 71 min, based on available data, more than the control group (57 min for imputed data). Alcohol intake, in terms of heavy episodic drinking and total weekly consumption, was lower in the intervention group with a probability of ≥ 75% for any intervention effect on alcohol intake. No clear evidence could be seen for an intervention effect on smoking cessation.

**Table 3:** Total effects of the intervention on primary outcomes at the 4-month interval

	Available data^1^		Imputed data^2^	
	Est. Mean (95% CI)	Pr.	Est. Mean (95% CI)	Pr.
**Fruit and vegetables** ^3^				
Intervention vs. control	0.19 (-0.05; 0.42)	94.0%	0.16 (-0.06; 0.39)	92.5%
**MVPA** ^**4**^				
Intervention vs. control	71.16 (15.72; 126.71)	99.4%	57.19 (3.66;110.99)	98.2%
	**Est. IRR (95% CI)**	**Pr.**	**Est. IRR (95% CI)**	**Pr.**
**Sugary drinks** ^**5**^				
Intervention vs. control	1.01 (0.82;1.24)	52.6%	0.98 (0.80;1.20)	56.8%
**Heavy episodic drinking** ^**6**^				
Intervention vs. control	0.85 (0.60;1.22)	80.9%	0.89 (0.64;1.24)	75.8%
**Weekly alcohol consumption** ^**7**^				
Intervention vs. control	0.81 (0.49;1.35)	78.5%	0.86 (0.54;1.35)	75.0%
	**Est. OR (95% CI)**	**Pr.**	**Est. OR (95% CI)**	**Pr.**
**Smoking cessation** ^**8**^				
Intervention vs. control	1.01 (0.62;1.66)	52.4%	1.08 (0.71;1.65)	63.5%

Analyses include all trial participants, regardless of presence or absence of risk behaviors for the analyzed lifestyle behavior

^1^Available data intervention/control: Fruit and vegetables *n* = 180/241; MVPA *n* = 180/241; sugary drinks *n* = 180/239; heavy episodic drinking *n* = 183/247; total weekly alcohol consumption *n* = 183/248; smoking cessation *n* = 182/244

^2^Imputed data intervention/control *n* = 377/379

Intervention effect on: ^3^daily portions (á 100 g) of fruit and vegetables consumed the past week; ^4^minutes of MVPA the past week;^5^units (33 cl) of sugary drinks the past week; ^6^number of times consuming > 4 standard drinks of alcohol the past month; ^7^number of standard drinks of alcohol the past week; ^8^smoking cessation (yes/no) the past month

Est.– Median of the marginal posterior distribution of adjusted mean/IRR/OR

CI– Compatibility interval (defined by the 2.5% and 97.5% percentiles of the posterior distribution)

Pr.– Proportion of the posterior distribution above or below the null, in the direction of the point estimate

MVPA– moderate-to-vigorous physical activity; IRR– incidence rate ratios; OR– Odds ratios

### Natural direct and natural indirect effects

Estimates of natural indirect effects and natural direct effects of group allocation on health behaviors are presented in Tables 4, 5, 6, 7, 8 and 9 including analyses based on available- and imputed data. Minutes in MVPA and portions of weekly fruit- and vegetables- intake are presented as estimated mean differences. Outcomes for heavy episodic drinking and total weekly consumption of alcohol and sugary drinks are expressed as incidence rate ratios (IRRs), while smoking cessation is expressed as odds ratios (ORs).

### Physical activity

Estimates of natural direct and natural indirect effects on physical activity, in terms of minutes spent in MVPA the last week, are presented in Table 4. The results show that the intervention’s effect on MVPA was partially mediated by increasing participants confidence; however, a substantial natural direct effect remained indicating that most of the intervention’s effect could not be explained by improvements in confidence. Further, changes in importance and know-how had a negligible impact on increased MVPA. Available- and imputed- data resulted in findings in the same direction with smaller effects based on imputed data.

**Table 4:** Natural indirect- and direct effects of intervention on minutes of moderate-to-vigorous physical activity

MVPA	2-month mediator -> 4-month outcome			
	Available data^1^		Imputed data^2^	
	Est. Mean (95% CI)	Pr.	Est. Mean (95% CI)	Pr.
**Confidence**				
Natural indirect effect	8.21 (0.01; 20.24)	97.5%	5.27 (-0.44; 15.52)	96.1%
Natural direct effect	72.87 (16.35; 129.39)	99.5%	51.25 (-2.3; 104.49)	96.9%
**Importance**				
Natural indirect effect	-1.34 (-8.73; 3.89)	73.4%	-1.28 (-7.87; 2.31)	78.5%
Natural direct effect	79.95 (22.1; 138.11)	99.6%	60.48 (6.89; 114.02)	98.7%
**Know-how**				
Natural indirect effect	3.03 (-3.91; 12.49)	82.3%	1.08 (-6.57; 10.12)	63.4%
Natural direct effect	77.49 (19.65; 134.72)	99.6%	55.89 (1.24; 110.34)	97.8%
**Combined**				
Natural indirect effect	5.23 (-8.55; 19.89)	78.4%	2.74 (-10.48; 16.79)	67.2%
Natural direct effect	76.34 (19.37; 134.16)	99.5%	55.22 (0.87; 109.7)	97.7%

^1^Available data intervention/control: MVPA *n* = 180/241, mediators *n* = 237/274

^2^Imputed data intervention/control *n* = 377/379

Est.– Median of the marginal posterior distribution of adjusted means

CI– Compatibility interval (defined by the 2.5% and 97.5% percentiles of the posterior distribution)

Pr.– Proportion of the posterior distribution above or below the null, in the direction of the point estimate

MVPA– moderate-to-vigorous physical activity

### Diet

Estimates of natural direct and indirect effect on intake of fruit and vegetables and sugary drinks are presented in Tables 5 and 6. The combined mediator models (available and imputed data) showed that the effect of the intervention on fruit and vegetable intake was partially explained by the mediators. For single mediators, importance did not explain any of the effect. The evidence suggested that weekly consumption of sugary drinks was not affected by the intervention, and no strong evidence of any mediated effects were found.

### Alcohol

Estimates of natural direct and indirect effect on heavy episodic drinking and total weekly alcohol consumption are presented in Tables 7 and 8. The results show a high probability of an intervention effect on a decreased frequency of heavy episodic drinking and a decreased total weekly alcohol consumption. However, the evidence also indicated that the effects could not be explained by changes in any of the mediators.

**Table 5:** Natural indirect- and direct effects of intervention on daily portions (á 100 g) of fruit and vegetables

Fruit and vegetables	2-month mediator -> 4-month outcome			
	Available data^1^		Imputed data^2^	
	Est. Mean (95% CI)	Pr.	Est. Mean (95% CI)	Pr.
**Confidence**				
Natural indirect effect	0.03 (-0.0; 0.08)	97.0%	0.01 (-0.01; 0.05)	88.9%
Natural direct effect	0.2 (-0.05; 0.46)	93.8%	0.16 (-0.06; 0.39)	92.2%
**Importance**				
Natural indirect effect	-0.01 (-0.04; 0.02)	72.9%	0.0 (-0.03; 0.01)	72.5%
Natural direct effect	0.24 (-0.02; 0.5)	96.3%	0.19 (-0.03; 0.41)	95.4%
**Know-how**				
Natural indirect effect	0.03 (0.0; 0.08)	97.6%	0.01 (-0.01; 0.05)	83.8%
Natural direct effect	0.19 (-0.07; 0.45)	92.1%	0.14 (-0.08; 0.37)	89.8%
**Combined**				
Natural indirect effect	0.04 (-0.01; 0.1)	93.7%	0.02 (-0.02; 0.06)	80.4%
Natural direct effect	0.19 (-0.08; 0.45)	91.9%	0.15 (-0.08; 0.37)	90.1%

^1^Available data intervention/control: Fruit and vegetables *n* = 180/241, mediators *n* = 237/274

^2^Imputed data intervention/control *n* = 377/379

Est.– Median of the marginal posterior distribution of adjusted means

CI– Compatibility interval (defined by the 2.5% and 97.5% percentiles of the posterior distribution)

Pr.– Proportion of the posterior distribution above or below the null, in the direction of the point estimate

**Table 6:** Natural indirect- and direct effects of intervention on weekly consumption of cans (á 33 cl) of sugary drinks

Sugary drinks	2-month mediator -> 4-month outcome			
	Available data^1^		Imputed data^2^	
	Est. IRR (95% CI)	Pr.	Est. IRR (95% CI)	Pr.
**Confidence**				
Natural indirect effect	0.99 (0.96; 1.01)	90.1%	0.99 (0.96; 1.01)	86.7%
Natural direct effect	1.05 (0.85; 1.3)	65.9%	0.98 (0.80; 1.20)	59.3%
**Importance**				
Natural indirect effect	1.0 (0.99; 1.01)	52.0%	1.0 (0.99; 1.02)	61.0%
Natural direct effect	1.03 (0.83; 1.29)	62.5%	0.97 (0.79; 1.19)	63.0%
**Know-how**				
Natural indirect effect	1.02 (1.0; 1.06)	94.0%	1.02 (0.99; 1.06)	92.7%
Natural direct effect	1.03 (0.83; 1.28)	59.6%	0.97 (0.79; 1.18)	63.0%
**Combined**				
Natural indirect effect	1.01 (0.95; 1.07)	63.5%	1.01 (0.96; 1.07)	67,5%
Natural direct effect	1.03 (0.83; 1.28)	62.4%	0.97 (0.79; 1.18)	62.7%

^1^Available data intervention/control: Sugary drinks *n* = 180/239, mediators *n* = 237/274

^2^Imputed data intervention/control *n* = 377/379

Est.– Median of the marginal posterior distribution of adjusted IRR (incidence rate ratios)

CI– Compatibility interval (defined by the 2.5% and 97.5% percentiles of the posterior distribution)

Pr.– Proportion of the posterior distribution above or below the null, in the direction of the point estimate

### Smoking cessation

Estimates of natural direct and natural indirect effects of smoking cessation (Table 9) show that the effect of the intervention on increased smoking cessation could not be explained by any of the mediators.

**Table 7:** Natural indirect- and direct effects of intervention on frequency of heavy episodic drinking

Heavy episodic drinking	2-month mediator -> 4-month outcome			
	Available data^1^		Imputed data^2^	
	Est. IRR (95% CI)	Pr.	Est. IRR (95% CI)	Pr.
**Confidence**				
Natural indirect effect	0.99 (0.94; 1.02)	83.5%	0.98 (0.94; 1.01)	85.8%
Natural direct effect	0.68 (0.47; 0.99)	97.9%	0.90 (0.65; 1.24)	74.2%
**Importance**				
Natural indirect effect	1.01 (0.97; 1.07)	73.5%	1.02 (0.98; 1.08)	81.6%
Natural direct effect	0.64 (0.44; 0.93)	99.1%	0.82 (0.59; 1.14)	88.1%
**Know-how**				
Natural indirect effect	1.01 (0.96; 1.07)	72.6%	0.99 (0.94; 1.05)	65.3%
Natural direct effect	0.69 (0.48; 1.01)	97.2%	0.91 (0.66; 1.27)	70.6%
**Combined**				
Natural indirect effect	1.04 (0.97; 1.15)	87.6%	1.02 (0.95; 1.11)	73.2%
Natural direct effect	0.65 (0.45; 0.95)	98.6%	0.84 (0.60; 1.18)	84.4%

^1^Available data intervention/control: Heavy episodic drinking *n* = 183/247, mediators *n* = 237/274

^2^Imputed data intervention/control *n* = 377/379

Est.– Median of the marginal posterior distribution of adjusted IRR (incidence rate ratios)

CI– Compatibility interval (defined by the 2.5% and 97.5% percentiles of the posterior distribution)

Pr.– Proportion of the posterior distribution above or below the null, in the direction of the point estimate

**Table 8:** Natural indirect- and direct effects of intervention on total weekly alcohol consumption

Total weekly alcohol consumption	2-month mediator -> 4-month outcome			
	Available data^1^		Imputed data^2^	
	Est. IRR (95% CI)	Pr.	Est. IRR (95% CI)	Pr.
**Confidence**				
Natural indirect effect	1.0 (0.94; 1.05)	44.4%	0.99 (0.94; 1.03)	75.1%
Natural direct effect	0.68 (0.4; 1.16)	91.9%	0.85 (0.54; 1.32)	77.0%
**Importance**				
Natural indirect effect	1.01 (0.97; 1.08)	74.2%	1.02 (0.98; 1.09)	82.2%
Natural direct effect	0.72 (0.43; 1.23)	88.8%	0.83 (0.52; 1.30)	79.5%
**Know-how**				
Natural indirect effect	1.0 (0.92; 1.09)	50.4%	0.98 (0.92; 1.05)	72.6%
Natural direct effect	0.69 (0.41; 1.17)	91.9%	0.86 (0.55; 1.34)	75.5%
**Combined**				
Natural indirect effect	1.05 (0.94; 1.21)	81.5%	1.03 (0.94; 1.16)	73.2%
Natural direct effect	0.72 (0.42; 1.24)	88.9%	0.83 (0.52; 1.31)	78.9%

^1^Available data intervention/control: Total weekly alcohol consumption *n* = 183/248, mediators *n* = 237/274

^2^Imputed data intervention/control *n* = 377/379

Est.– Median of the marginal posterior distribution of adjusted IRR (incidence rate ratios)

CI– Compatibility interval (defined by the 2.5% and 97.5% percentiles of the posterior distribution)

Pr.– Proportion of the posterior distribution above or below the null, in the direction of the point estimate

**Table 9:** Natural indirect- and direct effects of intervention on smoking cessation

Smoking cessation	2-month mediator -> 4-month outcome			
	Available data^1^		Imputed data^2^	
	Est. OR (95% CI)	Pr.	Est. OR (95% CI)	Pr.
**Confidence**				
Natural indirect effect	1.02 (0.98; 1.11)	86.0%	1.04 (1.0; 1.13)	96.0%
Natural direct effect	1.21 (0.69; 2.13)	74.7%	1.03 (0.68; 1.59)	55.7%
**Importance**				
Natural indirect effect	0.99 (0.93; 1.03)	71.6%	0.98 (0.91; 1.03)	81.0%
Natural direct effect	1.26 (0.72; 2.24)	79.1%	1.11 (0.73; 1.72)	69.2%
**Know-how**				
Natural indirect effect	0.97 (0.88; 1.04)	82.9%	0.99 (0.92; 1.06)	62.1%
Natural direct effect	1.26 (0.72; 2.23)	79.3%	1.08 (0.71; 1.67)	64.5%
**Combined**				
Natural indirect effect	0.96 (0.83; 1.07)	78.4%	0.98 (0.86; 1.09)	67.1%
Natural direct effect	1.31 (0.74; 2.32)	82.9%	1.12 (0.73; 1.75)	69.1%

^1^Available data intervention/control: Smoking cessation *n* = 182/244, mediators *n* = 237/274

^2^Imputed data intervention/control *n* = 377/379

Est.– Median of the marginal posterior distribution of adjusted OR (odds ratios)

CI– Compatibility interval (defined by the 2.5% and 97.5% percentiles of the posterior distribution)

Pr.– Proportion of the posterior distribution above or below the null, in the direction of the point estimate

## Discussion

In this study we investigated the mediated effects of a digital intervention on multiple lifestyle behaviors in high school students with respect to confidence, importance, and know-how. The results show an intervention effect of the mediators, confidence and know-how, but there was no observed effect of importance. Further, the mediated effects varied among the different behavioral outcomes. For MVPA and fruit and vegetable intake we found evidence that the total effect could partially be explained by improvements in confidence and know-how; however, a substantial part of the effects could not be explained by the hypothesized mediators. For alcohol outcomes, there were total intervention effects for decreased consumption, but no mediation effects could be detected. Regarding smoking and consumption of sugary drinks, we found no evidence for either mediation or total effects.

The weekly increase of approximately one hour of MVPA was partly, to a small extent, mediated by confidence. According to studies in both adults and adolescents there is strong evidence of mediated effects of self-efficacy (i.e., confidence) on PA outcomes [43–46]. Nevertheless, an Australian study found that a digital school-based intervention increased PA level without involvement of psychosocial mediating effects, in terms of self-efficacy together with self-control, knowledge, and behavioral intentions [29]. These findings, together with our study results, show that improvements in PA level can occur without, or with small mediating effects from self-efficacy. Both our study and the study by O’Dean et al. [29] targeted multiple health behaviors. Therefore, it is likely that the content of these interventions for reinforcing confidence for PA behaviors could not be as extensive as interventions focused on PA alone [44–46]. This may have resulted in a decreased impact of self-efficacy as a mediator for MVPA. Our results further demonstrate a small mediation effect of know-how on increased weekly MVPA, which is in line with previous findings showing that skills training, e.g., decision making skills, resistance training skills, and self-regulation skills, reflective of know-how, are likely mediators of PA outcomes [44, 47, 48]. In a study by Smith et al. [47] a school-based PA intervention for adolescent boys targeted development of resistance training skills by having teachers explain, model, and give feedback on resistance training exercises. The intervention increased muscular fitness, and most of this effect was mediated by resistance training skills. In the LIFE4YOUth intervention know-how focused on several health behaviors and was therefore likely less specific to PA compared with the intervention by Smith et al. [47]. This may have decreased the impact of know-how as a mediator. It is also reasonable to hypothesize that improved levels of MVPA were mainly due to mediating factors that were not the focus of this study. Goal setting is a behavior change technique commonly targeted in interventions effectively increasing PA [49–51]. According to the Goal Setting Theory, specific and high goals can improve a person’s performance through mediation by constructs of motivation and individual action plans to attain the goal [52]. Although goal setting was a key component in the LIFE4YOUth intervention, we found no intervention effect on motivation, in terms of importance. Instead, it is possible that improved goal setting behavior could have increased weekly MVPA mediated through specific strategies such as action- or coping- planning, which is in line with findings from other physical activity interventions [53, 54], However, further research is needed to explore potentially undetected mediators.

For fruit and vegetable intake in our trial, both know-how and confidence had partial, small mediating effects. For confidence, extant studies in adolescents and adults have reported mediated effects on fruit and vegetable intake [55–57], while others have found that changes in self-efficacy are predictive of short-term changes in dietary behaviors [44]. The mediated effects of know-how on dietary behavior change have, to the best of our knowledge, not been previously evaluated in children and adolescents. Instead, factors closely related to know-how, such as skills training, are commonly included in interventions to improve dietary behaviors [44, 46, 55], but other behavioral factors, e.g., health routines, perceived behavioral control, and intention, are assessed as potential mediators for dietary changes [46, 55, 56, 58, 59]. Kothe et al. [60] evaluated fruit and vegetable intake in university students in Australia receiving e-mails with content based on the Theory of Planned Behavior. The authors found that perceived behavioral control was predictive of intention, which in turn predicted fruit and vegetable intake. Intention has also been found to have a mediation effect on fruit and vegetable intake among university students in China [56]. With this background, it is possible that the increased know-how for change in our trial may have raised control beliefs, which in turn improved perceived behavioral control, followed by intention, and thereafter fruit and vegetable consumption.

Regarding intake of sugary drinks, a trial in young adults [55] found that increased self-efficacy scores had significant mediation effects on decreased intake post-intervention at three months and at long-term follow-up at nine months. This contrasts with our results, which in line with O’Dean et al. [29], showing that for sugary drinks consumption among adolescents there was little evidence of any mediation or total effects. The difference in the intervention content between the trials may explain the disparity; Partridge et al. [55] used personalized content in the form of coaching calls with a dietitian, whilst the current study adopted a more generalist approach for both dietary behaviours and other health behaviours. Hence, the Partridge et al. [55] intervention was more resource intensive and focused specifically on dietary aspects. Moreover, it is likely that the contrasting findings are related to the study populations, where adolescents are likely to have greater barriers for behavior change due to e.g., peer pressure and emotionally driven decisions [19, 61]. Although peer effects and social norms are also important in behavior change in young adults, it is possible that adolescents may be even more sensitive to their social context [62, 63].

In contrast to our findings, previous literature in adults have shown clear mediation effects of confidence and know-how on reducing alcohol consumption and for smoking cessation [27, 28]. Findings from the multiple health behavior change intervention by O’Dean et al. [29] show that the mediated effects and the intervention effects for alcohol consumption and smoking took different directions. Mediators, in terms of knowledge, self-efficacy, intention, and self-control, had an impact on decreased consumption while the intervention itself seem to have increased the intake somewhat; something that may be related to that open discussions about alcohol and smoking could potentially have primed some adolescents to increase alcohol intake and smoking frequency [29]. Neither of the findings above were replicated among the Swedish high school students in this trial. A possible explanation for the lack of mediated effects on smoking cessation and alcohol intake could be related to that we used mediation questions connected to overall lifestyle behaviors rather than linked with the specific areas of alcohol, smoking, PA, and diet. It is likely that the mediation questions were therefore more reflective of PA and dietary behavior, since it may be more intuitive among high school students to primarily connect a ‘healthy lifestyle’ with these areas before thinking about alcohol and smoking. Another possible reason for the contrasting findings, as outlined by the Prototype Willingness Model [61], could be related to differences between how the students perceived a person (i.e., a prototype) who smokes or drinks alcohol, and how willing they were to (not) smoke or drink. The intervention did not target these factors, but this social reaction part of the model, that accounts for how social reaction impacts behavior (i.e., drinking or smoking because your peers are doing it), has been shown to predict adolescent alcohol consumption and smoking [64, 65], and has been demonstrated to have a greater impact on adolescent behavior than adult behavior [66].

In contrast to confidence and know-how, we did not see an intervention effect on importance, which is in line with interventions targeting smoking cessation and reduced alcohol consumption in adults [27, 28]. However, in the studies by Bendtsen [27] and Crawford [28] the adults rated importance as very high at baseline compared with the adolescents in our study. According to the Social Cognitive Theory [19], finding the goal important is a cornerstone to successfully change a behavior. Therefore, lack of importance or lack of motivation is a barrier to creating a healthy lifestyle behavior. Further, importance has been suggested as a factor that predicts readiness to change [35]. This implicate that the high school students, on average, may have been less ready to improve lifestyle behaviors compared with previous studies in adults [28, 31]. However, there are great individual variations in what aspects, e.g., social concerns and cost-related aspects, that affect motivation and how high adolescents rate readiness to create healthy lifestyle behaviors [67–69]. In a transtheoretical model dietary intervention [69], readiness to change [70],, was found to be an evident mediator of fruit and vegetable intake among adolescents taking part of a dietary program designed for youths in different stages of change. It is therefore possible that interventions promoting healthy lifestyle choices for adolescents would gain from a design that allows for tailored strategies based on e.g., different stages of readiness to change. Another aspect to consider and further evaluate, is if the mediation effects of importance, confidence, and know-how are different, and more evident, when focused on targeting fewer lifestyle behaviors. It has been suggested that refraining from smoking and alcohol consumption versus increasing healthy dietary intake and PA levels, comprise different behavioral processes [71]. It is therefore possible that it is more difficult to evaluate, and potentially achieve, clear improvements of behavior change outcomes and mediated effects when multiple behavior change is targeted. Since risk behaviors seem to appear in clusters [16, 72] it is possible that the same thing goes for improvements of behavior, i.e., if a person improves one lifestyle aspect it may spill over on another.

When evaluating the total intervention effect in this study, it was found that the intervention group consumed approximately 19 g more of fruit and vegetables daily compared to the control group, which is comparable to one additional apple per week. For physical activity, the intervention group engaged in 71 min more MVPA each week compared to the control group, which can be translated to a brisk walk 10 min daily. Hence, the total intervention effect for daily MVPA had a more evident clinical significance compared to the daily fruit and vegetable intake. Both increased PA levels as well as intake of fruit and vegetables are associated with several health aspects in adolescents [73–75] and it is likely that individuals with the unhealthiest dietary habits and the most sedentary lifestyle can achieve the greatest health benefits from increasing these health behaviors [76, 77]. However, it is beyond the scope of this study to speculate on the health benefits on a specific level for the included participants.

Limitations.

There are several limitations that should be borne in mind when interpreting the results from this study. As mentioned previously, questions for the assessed mediators about a ‘healthy lifestyle’ may not have been equally reflective of all behaviors targeted by the intervention. Moreover, self-reported data leads to a higher uncertainty about the actual lifestyle behavior, and objective measurement such as assessing MVPA with accelerometry would have increased the specificity of the findings. However, the pragmatic and naturalistic design of the study prohibited objective measures, and the brief self-reported questions were specifically chosen to reduce participant burden and increase response rate. It is likely that attrition would have been even higher if more comprehensive measures had been used. The study findings need to be interpreted with caution given the high attrition rate and that participants from high socioeconomic groups were overrepresented. The current mediation evaluation is based on data from an RCT assuring causality for the intervention-outcome and intervention-mediator. However, the relationship between mediator-outcome is potentially confounded and although we adjusted for baseline characteristics the results could be biased.

## Conclusions

This study investigated the mediated effects of LIFE4YOUth– a digital intervention targeting multiple lifestyle behaviors among high school students in Sweden. Improvements in weekly MVPA and daily fruit and vegetables intake were to a small extent mediated by confidence and know-how, but mainly a result of other unknown factors. No mediated effects were seen for smoking, alcohol- or sugary drinks intake. Different strategies for assessing psychosocial mediators in studies evaluating multiple health behavior change in adolescents should be further explored.

## Electronic supplementary material

Below is the link to the electronic supplementary material.

Supplementary Material 1

Supplementary Material 2

## Data Availability

A study protocol, including a statistical analysis plan, is available open-access. Deidentified datasets generated during and/or analyzed during the current study will be made available upon reasonable request to MB, after approval of a proposal and with a signed data access agreement.

## Abbreviations

CI: Compatibility interval
IRR: Incidence rate ratio
MVPA: Moderate-to-vigorous-physical-activity
PA: Physical activity
RCT: Randomized controlled trial
OR: Odds ratio
