# Supplementary material for: Mediated effects of LIFE4YOUth—a mobile health intervention for multiple lifestyle behavior change among high school students in Sweden: findings from a randomized controlled trial
Source: BMC Public Health. 2025 Mar 7;25:922. doi: 10.1186/s12889-025-22097-5 (PMC11889938; doi:10.1186/s12889-025-22097-5)
Supplement: Supplementary file 1 — Supplementary Material 1 [file 12889_2025_22097_MOESM1_ESM.docx]

# Appendix A - Questionnaires

## Baseline questionnaire

1. Sex:
   1. Female
   2. Male
2. Age (numerical measure)
3. Where were you born?
   1. Sweden
   2. Other Nordic countries
   3. Other European country
   4. Country outside Europe
4. Where were your parents born?
   1. Sweden
   2. Other Nordic countries
   3. Other European country
   4. Country outside Europe
5. How would you describe the economic situation in your family?
   1. Very good
   2. Average
   3. Not so good
   4. Not good at all
6. Please select the highest education for your mother and father.
   1. Primary education
   2. Secondary education
   3. University education
7. Thinking about your own life and personal circumstances, how satisfied are you with your life as a whole?” with a 11-point scale from 0 (not at all satisfied) to 10 (completely satisfied).
8. How many standard drinks of alcohol did you consume last week? (numerical measure)
9. How often, during the past month, have you consumed four or more standard drinks of alcohol on one occasion? (numerical measure)
10. How many cigarettes did you smoke last week? (numerical measure)
11. How much time in total did you spend on moderate physical activity (e.g. bicycling or walking for transport or leisure) **last week**?
    1. 0
    2. Less than 30 minutes
    3. 30-60 minutes
    4. 1 hours
    5. 1.5 hours
    6. 2 hours
    7. 2.5 hours
    8. 3 hours
    9. 3.5 hours (i.e. 30 minutes per day)
    10. 4 hours
    11. 5 hours
    12. 6 hours
    13. 7 hours (i.e. 1 hour per day)
    14. 10.5 hours (i.e. 1.5 hours per day)
    15. 14 hours (i.e. 2 hours per day)
12. How much time in total did you spend on vigorous physical activity (i.e. activities producing fast increases in breathing or heart rate), for instance running, aerobics, etc. **last week?**
    1. 0
    2. Less than 30 minutes
    3. 30-60 minutes
    4. 1 hours
    5. 1.5 hours
    6. 2 hours
    7. 2.5 hours
    8. 3 hours
    9. 3.5 hours (i.e. 30 minutes per day)
    10. 4 hours
    11. 5 hours
    12. 6 hours
    13. 7 hours (i.e. 1 hour per day)
    14. 10.5 hours (i.e. 1.5 hours per day)
    15. 14 hours (i.e. 2 hours per day)
13. How many 100g portions (equivalent to an average sized banana or one large apple) of fruit did you consume **last week**?
    1. 0/0
    2. 1-2 portions **per week**/0.2
    3. 3-4 portions **per week/0.5**
    4. 5-6 portion **per week/0.8**
    5. 1.0 portion **per day/1**
    6. 1.5 portions **per day/1.5**
    7. 2.0 portions **per day**
    8. 2.5 portions **per day**
    9. 3.0 portions **per day or more**
14. How many 100 g portions (equivalent to an average handful) of vegetables did you consume **last week**?
    1. 0 (0)
    2. 1-2 portions **per week (0.2)**
    3. 3-4 portions **per week (0.5)**
    4. 5-6 portion **per week** (0.8)
    5. 1.0 portion **per day (1)**
    6. 1.5 portions **per day**
    7. 2.0 portions **per day**
    8. 2.5 portions **per day**
    9. 3.0 portions **per day or more**
15. How many cans (33 cl, one standard can) of sugary drinks (e.g. soft drinks, “energy drinks”) did you consume **last week**?
    1. 0 cans
    2. 1 can **per week**
    3. 2-3 cans **per week**
    4. 4-6 cans **per week**
    5. 1 can **per day**
    6. 1.5 cans **per day**
    7. 2.0 cans **per day**
    8. 2.5 cans **per day**
    9. 3.0 cans **per day or more**
16. How many portions of candy, chocolate, pastry (e.g. buns, muffins, cookies), ice cream and salty snacks (e.g. crisps, nuts, chees doodles) did you eat **last week**? *One portion is 50 g candy (9 pieces), 40 g chocolate (6 pieces/squares), 1 bun, 2 dl (scoops) of ice cream or 2 dl snacks (40 g)*.
    1. 0 portions 0
    2. 1 portion **per week**
    3. 2-3 portions **per week**
    4. 4-6 portions **per week**
    5. 1 portion **per day**
    6. 1.5 portions **per day**
    7. 2.0 portions **per day**
    8. 2.5 portions **per day**
    9. 3.0 portions **per day**
    10. 3.5 portions **per day**
    11. 4.0 portions **per day or more**
17. How tall are you? (in cm, numerical measure)
18. What is your current body weight? (in kg, numerical measure)
19. How important is it for you to improve your lifestyle behaviours? (10-point scale ranging from 1 = “Not important” to 10 = “Very important”)
20. How confident are you that you will be able to improve your lifestyle behaviours? (10-point scale ranging from 1 = “Not at all” to 10 = “Very confident”)
21. To what degree do you have the know-how and strategies to improve your lifestyle behaviours? (10-point scale ranging from 1 = “Not at all” to 10 = “Very high degree”)

**Note:** Participants are reminded of the definition of a standard unit of alcohol by graphical means, as well as given visual cues for what constitutes a portion of fruit, vegetables and a unit of sugary drinks.

## Follow-up questionnaire (Two- and four-month follow-up)

1. How many standard drinks of alcohol did you consume last week? (numerical measure)
2. How often, during the past month, have you consumed four or more standard drinks of alcohol on one occasion? (numerical measure)
3. Have you smoked any cigarettes the past four weeks?
   1. Yes
   2. No
4. (Smokers only) How many cigarettes did you smoke last week? (numerical measure)
5. How much time in total did you spend on moderate physical activity (e.g. bicycling or walking for transport or leisure) **last week**?
   1. 0
   2. Less than 30 minutes
   3. 30-60 minutes
   4. 1 hours
   5. 1.5 hours
   6. 2 hours
   7. 2.5 hours
   8. 3 hours
   9. 3.5 hours (i.e. 30 minutes per day)
   10. 4 hours
   11. 5 hours
   12. 6 hours
   13. 7 hours (i.e. 1 hour per day)
   14. 10.5 hours (i.e. 1.5 hours per day)
   15. 14 hours (i.e. 2 hours per day)
6. How much time in total did you spend on vigorous physical activity (i.e. producing fast increases in breathing or heart rate), for instance running, aerobics, etc. **last week?**
   1. 0
   2. Less than 30 minutes
   3. 30-60 minutes
   4. 1 hours
   5. 1.5 hours
   6. 2 hours
   7. 2.5 hours
   8. 3 hours
   9. 3.5 hours (i.e. 30 minutes per day)
   10. 4 hours
   11. 5 hours
   12. 6 hours
   13. 7 hours (i.e. 1 hour per day)
   14. 10.5 hours (i.e. 1.5 hours per day)
   15. 14 hours (i.e. 2 hours per day)
7. How many 100g portions (equivalent to an average sized banana or one large apple) of fruit did you consume **last week**?
   1. 0
   2. 1-2 portions **per week**
   3. 3-4 portions **per week**
   4. 5-6 portion **per week**
   5. 1.0 portion **per day**
   6. 1.5 portions **per day**
   7. 2.0 portions **per day**
   8. 2.5 portions **per day**
   9. 3.0 portions **per day or more**
8. How many 100 g portions (equivalent to an average handful) of vegetables did you consume **last week**?
   1. 0
   2. 1-2 portions **per week**
   3. 3-4 portions **per week**
   4. 5-6 portion **per week**
   5. 1.0 portion **per day**
   6. 1.5 portions **per day**
   7. 2.0 portions **per day**
   8. 2.5 portions **per day**
   9. 3.0 portions **per day or more**
9. How many cans (33 cl, one standard can) of sugary drinks (e.g. soft drinks, “energy drinks”) did you consume **last week**?
   1. 0 cans
   2. 1 can **per week**
   3. 2-3 cans **per week**
   4. 4-6 cans **per week**
   5. 1 can **per day**
   6. 1.5 cans **per day**
   7. 2.0 cans **per day**
   8. 2.5 cans **per day**
   9. 3.0 cans **per day or more**
10. How many portions of candy, chocolate, pastry (e.g. buns, muffins, cookies), ice cream and salty snacks (e.g. crisps, nuts, chees doodles) did you eat **last week**? *One portion is 50 g candy (9 pieces), 40 g chocolate (6 pieces/squares), 1 bun, 2 dl (scoops) of ice cream or 2 dl snacks (40 g)*.
    1. 0 portions
    2. 1 portion **per week**
    3. 2-3 portions **per week**
    4. 4-6 portions **per week**
    5. 1 portion **per day**
    6. 1.5 portions **per day**
    7. 2.0 portions **per day**
    8. 2.5 portions **per day**
    9. 3.0 portions **per day**
    10. 3.5 portions **per day**
    11. 4.0 portions **per day or more**

1. What is your current body weight? (in kg, numerical measure)
2. How important is it for you to improve or maintain healthy lifestyle behaviours? (10-point scale ranging from 1 = “Not important” to 10 = “Very important”)
3. How confident are you that you will be able to improve or maintain healthy lifestyle behaviours? (10-point scale ranging from 1 = “Not at all” to 10 = “Very confident”)
4. To what degree do you have the know-how and strategies to improve or maintain healthy lifestyle behaviours? (10-point scale ranging from 1 = “Not at all” to 10 = “Very high degree”)
