# Supplementary material for: Mediated effects of LIFE4YOUth—a mobile health intervention for multiple lifestyle behavior change among high school students in Sweden: findings from a randomized controlled trial
Source: BMC Public Health. 2025 Mar 7;25:922. doi: 10.1186/s12889-025-22097-5 (PMC11889938; doi:10.1186/s12889-025-22097-5)
Supplement: Supplementary file 2 — Supplementary Material 2 [file 12889_2025_22097_MOESM2_ESM.docx]

## Attrition analysis

Total effect at 4 months among sub-sample with 2-month mediator outcome vs total effect for everyone with available data on behavioral outcomes at 4 months

|  | **Sub-sample^1^** | | **Available data^2^** | |
| --- | --- | --- | --- | --- |
|  | **Est. Mean (95% CI)** | **Pr.** | **Est. Mean (95% CI)** | **Pr.** |
| **Fruit and vegetables^3^** |  |  |  |  |
| Intervention vs. control | 0.221 (-0.04;0.48) | 95.4% | 0.19 (-0.05; 0.42) | 94.0% |
| **MVPA^4^** |  |  |  |  |
| Intervention vs. control | 80.1 (24.46;137.35) | 99.7% | 71.16 (15.72; 126.71) | 99.4% |
|  | **Est. IRR (95% CI)** | **Pr.** | **Est. IRR (95% CI)** | **Pr.** |
| **Sugary drinks^5^** |  |  |  |  |
| Intervention vs. control | 1.06 (0.85;1.31) | 68.5% | 1.01 (0.82;1.24) | 52.6% |
| **Heavy episodic drinking^6^** |  |  |  |  |
| Intervention vs. control | 0.7 (0.48;1.02) | 97.0% | 0.85 (0.60;1.22) | 80.9% |
| **Weekly alcohol consumption^7^** |  |  |  |  |
| Intervention vs. control | 0.72 (0.43;1.23) | 88.4% | 0.81 (0.49;1.35) | 78.5% |
|  | **Est. OR (95% CI)** | **Pr.** | **Est. OR (95% CI)** | **Pr.** |
| **Smoking cessation^8^** |  |  |  |  |
| Intervention vs. control | 1.25 (0.71;2.19) | 78.2% | 1.01 (0.62;1.66) | 52.4% |

^1^Sub-sample with reported mediators at 2 months: importance- n=514; confidence n=511; know-how n=511.
^2^ Available behavioral outcomes at 4 months: Fruit and vegetables n=421; MVPA n=421; sugary drinks n=419; heavy episodic drinking n=430; total weekly alcohol consumption n=431; smoking cessation n=426.
Intervention effect on: ^3^daily portions (á 100 g) of fruit and vegetables consumed the past week; ^4^minutes of MVPA the past week ;^5^units (33 cl) of sugary drinks the past week; ^6^number of times consuming >4 standard drinks of alcohol the past month; ^7^number of standard drinks of alcohol the past week; ^8^smoking cessation (yes/no) the past month
Est. – Median of the marginal posterior distribution of adjusted mean/IRR/OR
CI – Compatibility interval (defined by the 2.5% and 97.5% percentiles of the posterior distribution)
Pr. – Proportion of the posterior distribution above or below the null, in the direction of the point estimate.
MVPA – moderate-to-vigorous physical activity; IRR – incidence rate ratios; OR – Odds ratio
